# Supplementary figures and images for: When does a Lotka-Volterra model represent microbial interactions? Insights from in vitro nasal bacterial communities
Source: mSystems. 2023 Jun 6;8(3):e00757-22. doi: 10.1128/msystems.00757-22 (PMC10308948; doi:10.1128/msystems.00757-22)

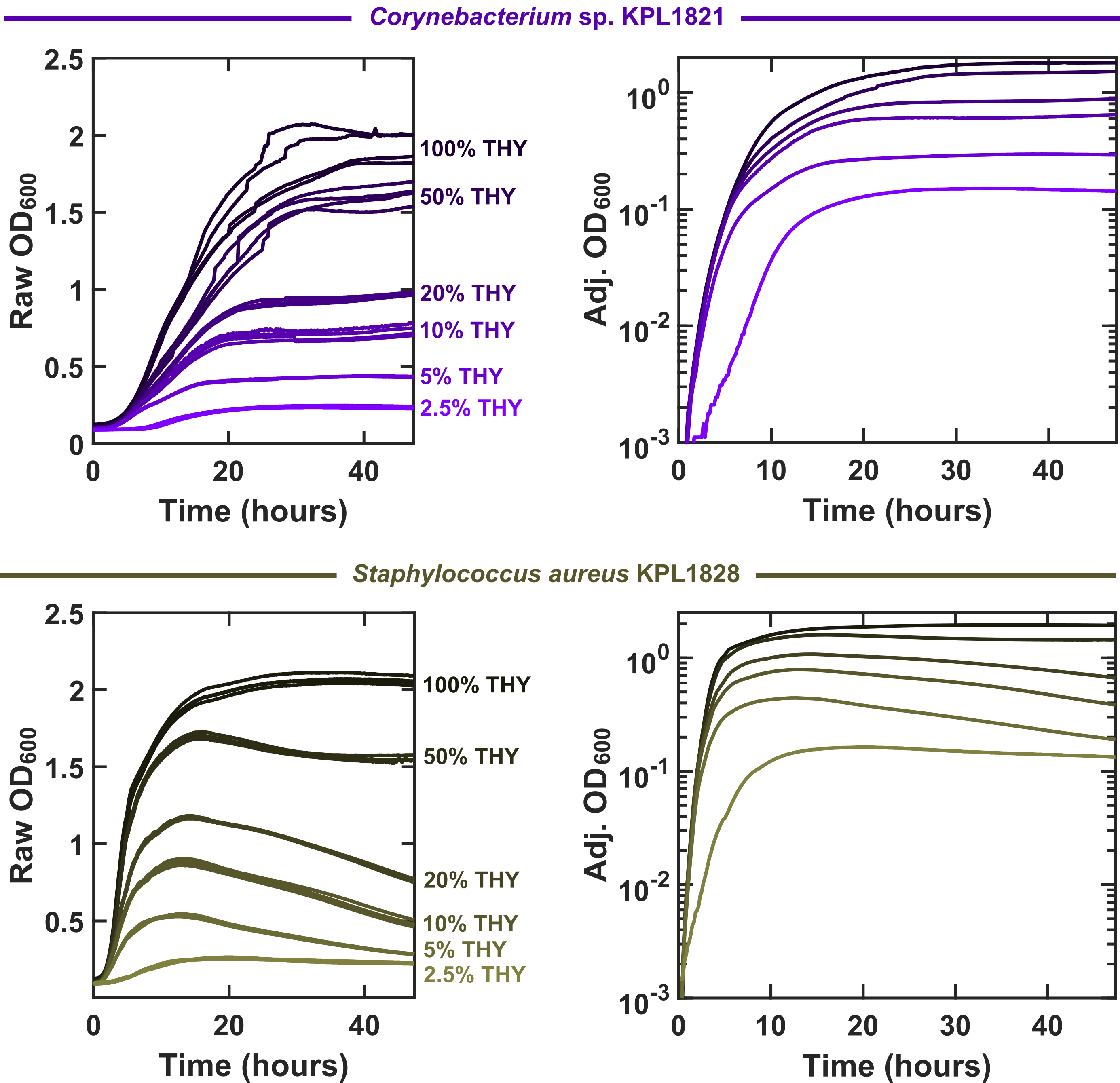

Supplement: Fig S1 — Growth rate and carrying capacity can be estimated by monitoring the OD600 of cultures over time. Growth curves of Corynebacterium sp. KPL1821 (top) and Staphylococcus aureus KPL1828 (bottom) are shown as two representative strains grown at different concentrations of THY (from 2.5% to 100%). For each strain, the plot on the left (four replicates) shows the raw OD600 values obtained from microplate reader and the plot on the right (average of four replicates) shows log-transformed values after subtracting the background (estimated as the average of the OD600 values measured within the first 30 min). Carrying capacities are estimated based on the maximum OD600 values within 48 h of growth (after adjustments described in Fig. S3). Growth rates are calculated by fitting a line into the log-transformed OD readings in early stages of growth (typically before OD600 reaches 30% of its maximum value). Four replicates are used in each case. [file msystems.00757-22-s0001.tif]

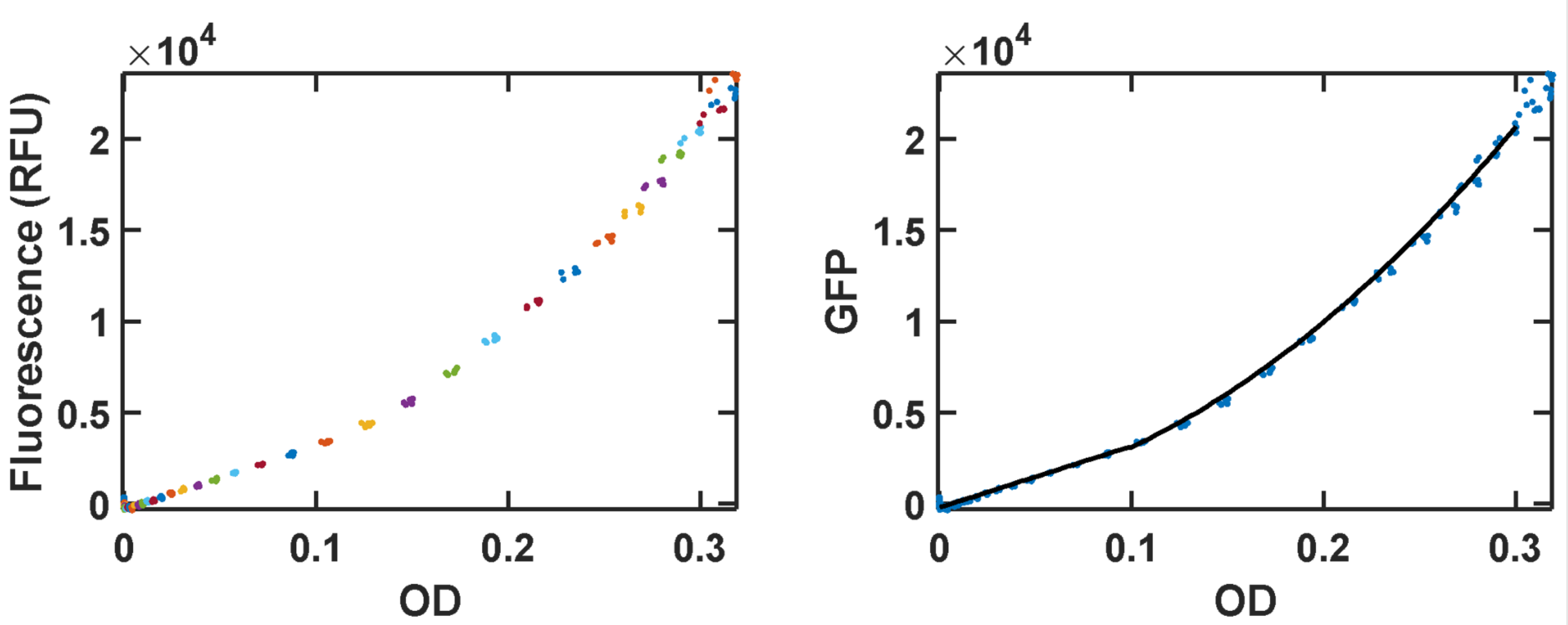

Supplement: Fig S2 — Fluorescence readings can be converted back to OD600 in sGFP Staphylococcus aureus cultures. Growth rate and fluorescence are tightly linked in growing cultures of sGFP S. aureus Newman (left). This relation can be used to derive a calibration curve that converts fluorescence readings to corresponding cell density (using OD as a proxy), as described by the equation in Materials and Methods (right). [file msystems.00757-22-s0002.tif]

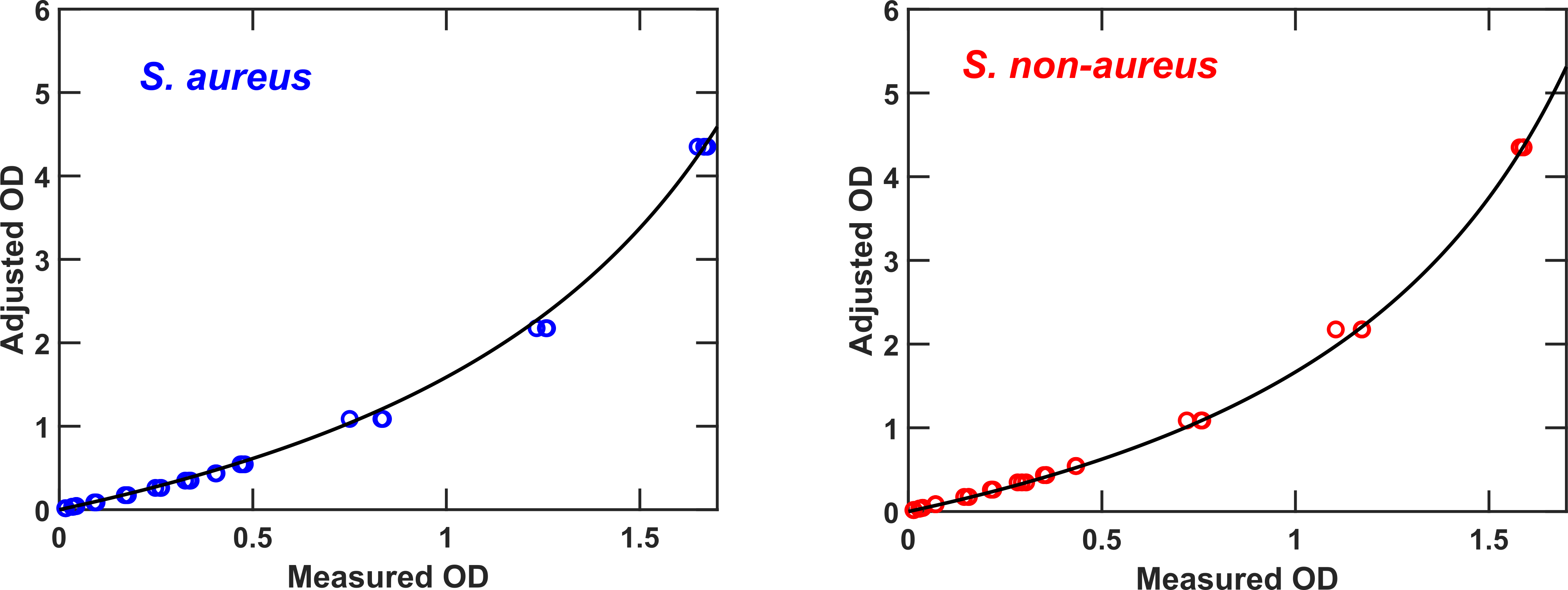

Supplement: Fig S3 — Measured OD600 can be used to estimate the cell density. Left: monoculture of Staphylococcus aureus. Right: monoculture of non-aureus Staphylococcus sp. KPL1850. Cells were grown to mid-exponential phase, concentrated to a high OD and then diluted back to lower ODs. Dilutions start from a measured OD of ~1.7 and cover the following dilution factors: 1, 0.5, 0.25, 0.125, 0.1, 0.08, 0.06, 0.04, 0.02, 0.01, 0.008, and 0.004. Three replicates were measured for each case. Samples diluted to an OD of 0.025 (within the linear range of OD-Density relation) were used to measure the CFUs, estimated around 1.6×10^9^ cells/mL at OD 1. The conversion equation between the measure OD and the adjusted OD (ODadj) is estimated to be OD adj= 2.7OD/(2.7-OD) for S. aureus and OD adj = 2.5OD/(2.5-OD) for non-aureus Staphylococcus sp. KPL1850. This correction is applied to adjust the OD when the measured OD was above 0.8 to more accurately estimate the interaction coefficients in cocultures. We have not adjusted the ODs in CFSM experiments (the measured OD600 is reported in those cases), because they did not affect any of our conclusions. [file msystems.00757-22-s0003.tif]

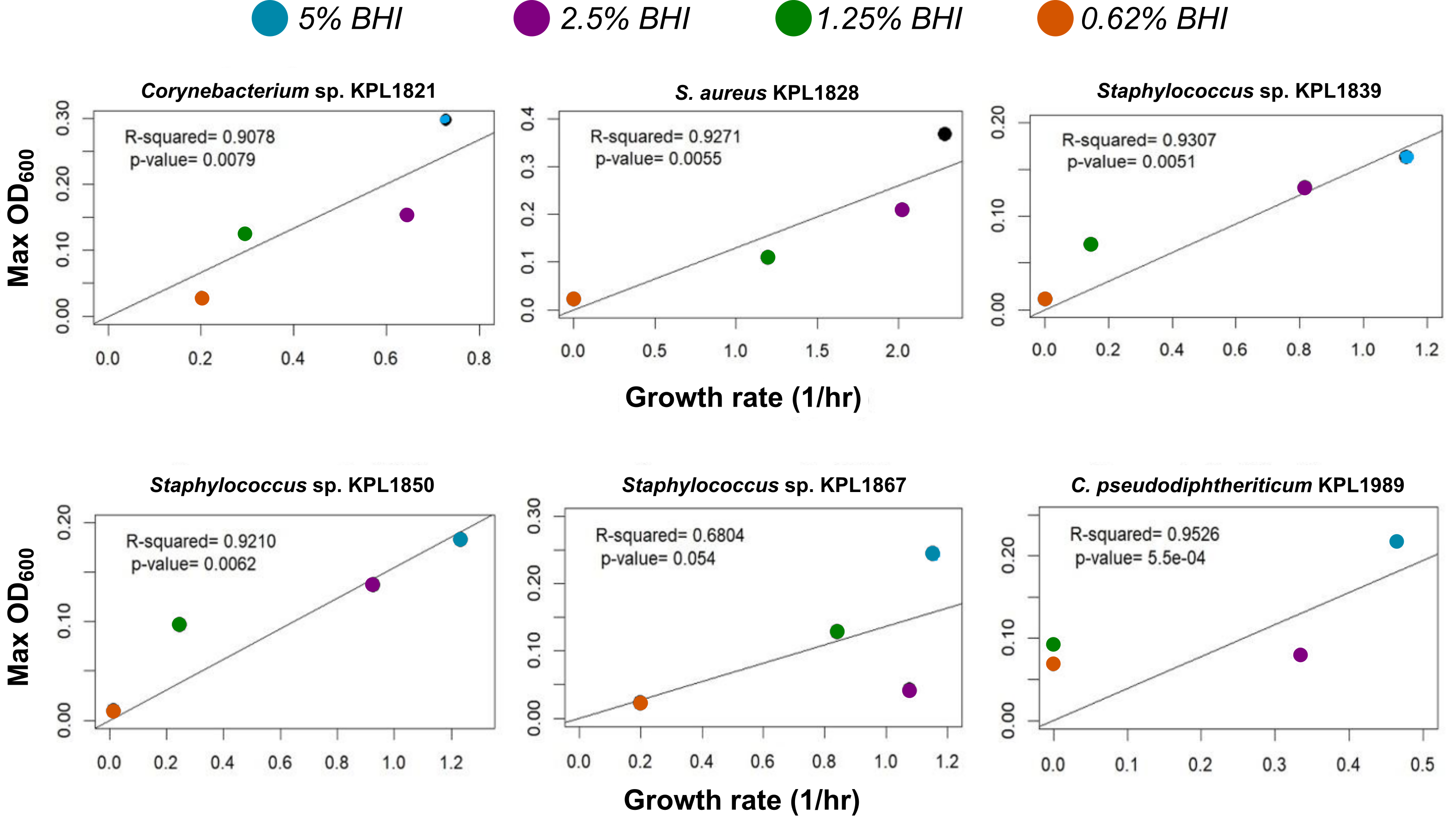

Supplement: Fig S4 — Growth rate-carrying capacity results from nasal isolates grown in low concentrations (5%–0.62%) of BHI show strong correlations. A linear regression analysis reveals a strong positive relationship between growth rate and carrying capacity when isolates are grown in low-nutrient concentrations. Each data point shows the average growth rate and carrying capacity (using Max OD600 as a proxy) from 3 to 6 technical replicates. [file msystems.00757-22-s0004.tif]

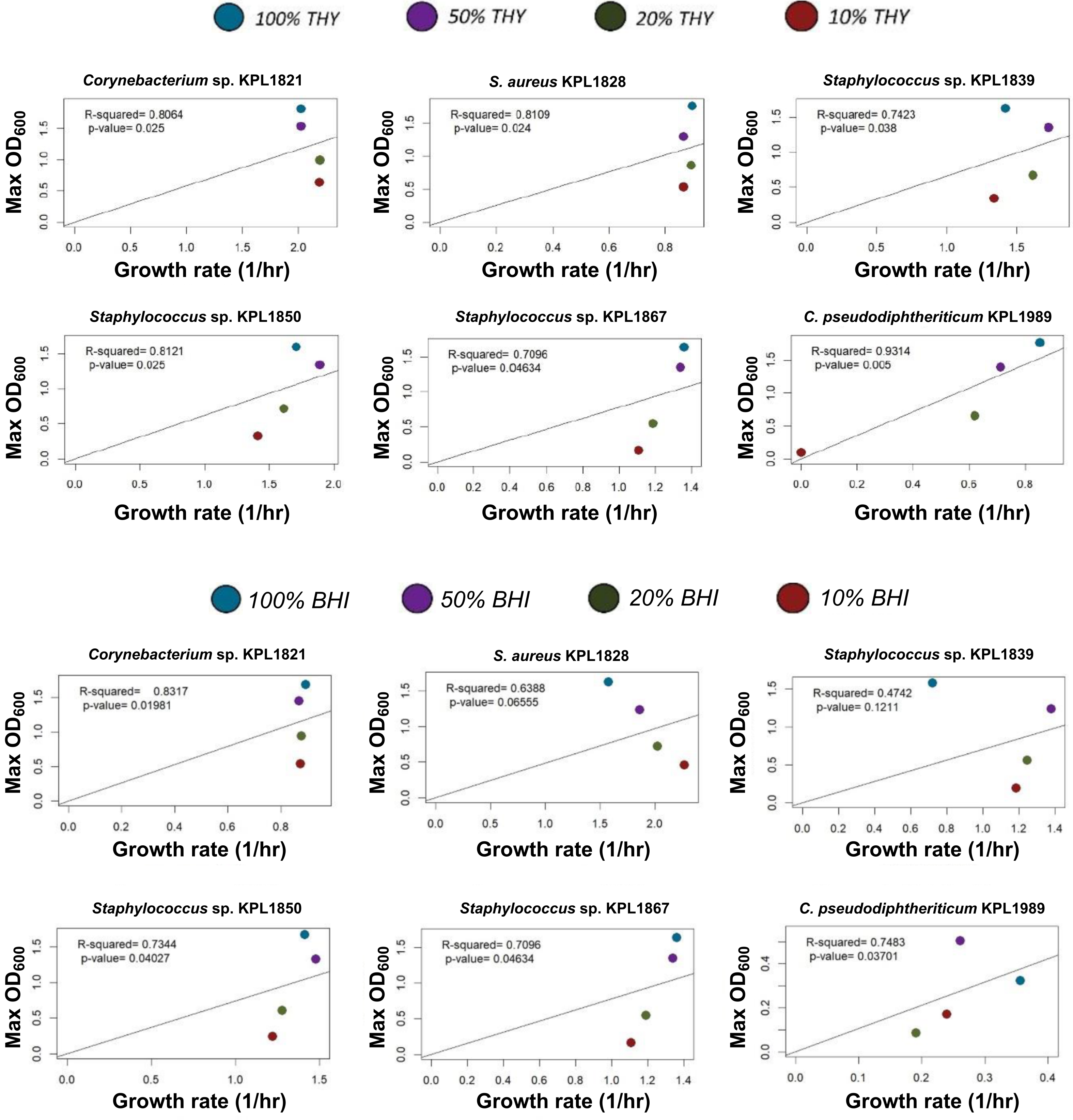

Supplement: Fig S5 — In high-nutrient environments, growth rate-carrying capacity relationships for nasal bacteria grown in different medium concentrations do not follow a simple proportionality relation. In high concentrations (100%–10%) of THY (top) and BHI (bottom) growth rate-carrying capacity relations deviate from a linear regression analysis calculated based on measured values at different concentrations. Each data point shows the average growth rate and carrying capacity (using Max OD600 as a proxy) from 3 to 6 technical replicates. [file msystems.00757-22-s0005.tif]

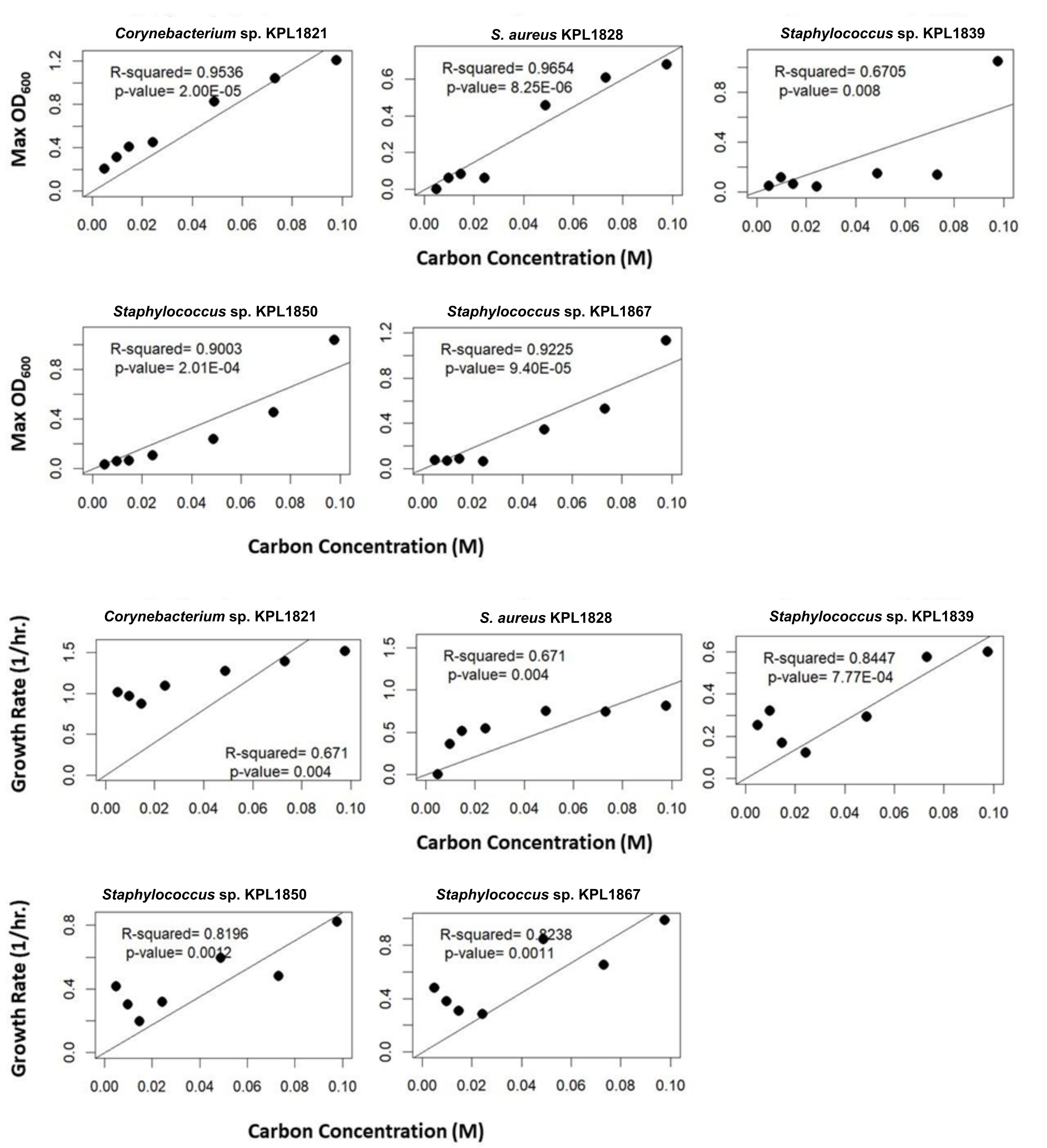

Supplement: Fig S6 — The carrying capacity of nasal isolates grown in a defined medium at various carbon concentrations is proportional to the total carbon concentration, but the growth rate is not. A linear regression analysis shows a positive correlation between carrying capacity and carbon concentrations (top). In contrast, a linear regression analysis shows only a moderate correlation between growth rate and carbon concentrations (bottom). Each data point shows the average growth rate and carrying capacity (using Max OD600 as a proxy) from 3 to 6 technical replicates from two independent experiments. Results for C. pseudodiphtheriticum KPL1989 were not consistent in different experiments and are not included here. [file msystems.00757-22-s0006.tif]

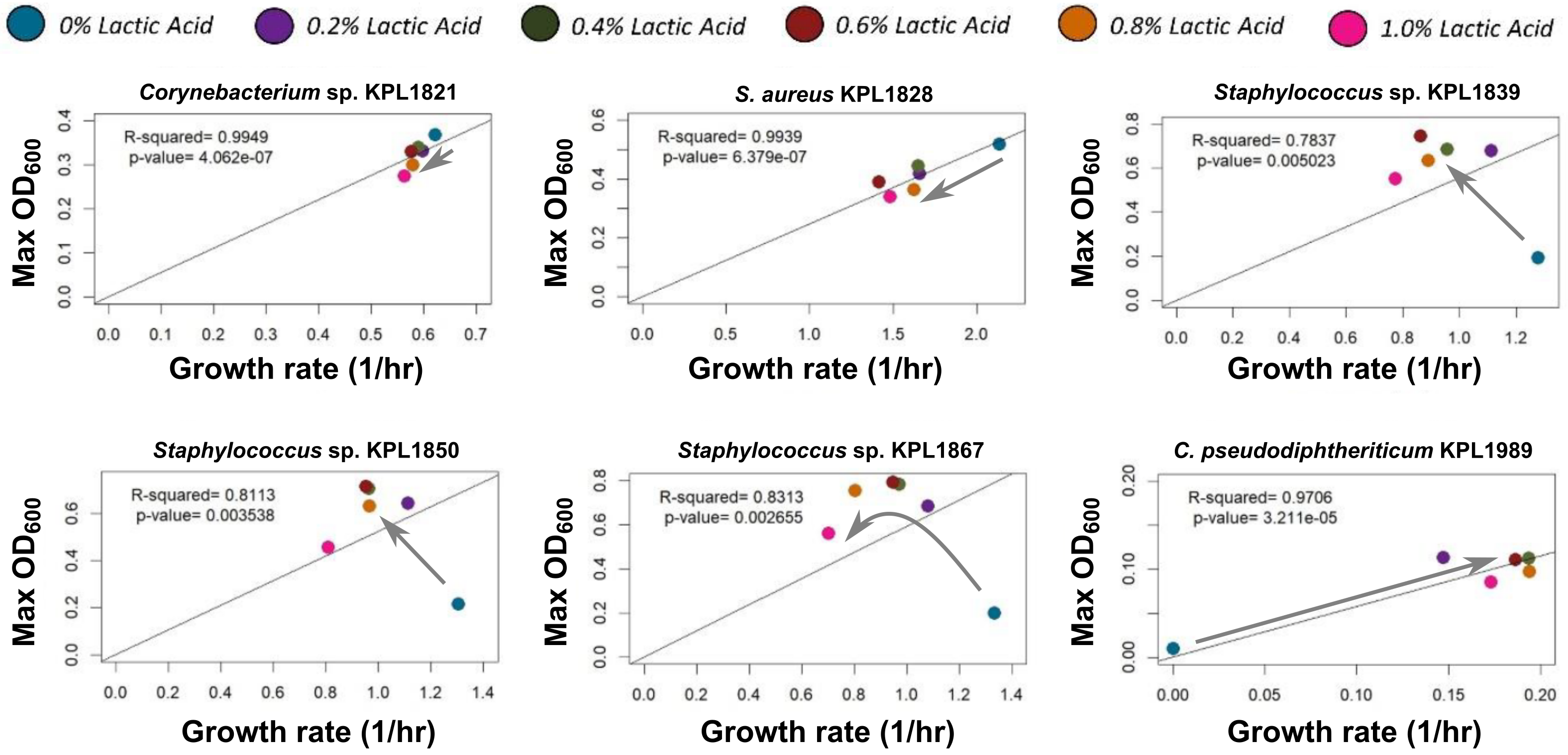

Supplement: Fig S7 — Growth rate-carrying capacity results from nasal isolates grown in the presence of lactic acid show different trends for different isolates. A linear regression analysis shows weak correlations between growth rate and carrying capacity. Each data point shows the average growth rate and carrying capacity (using Max OD600 as a proxy) from 3 to 6 technical replicates. Different trends are observed in different isolates. [file msystems.00757-22-s0007.tif]

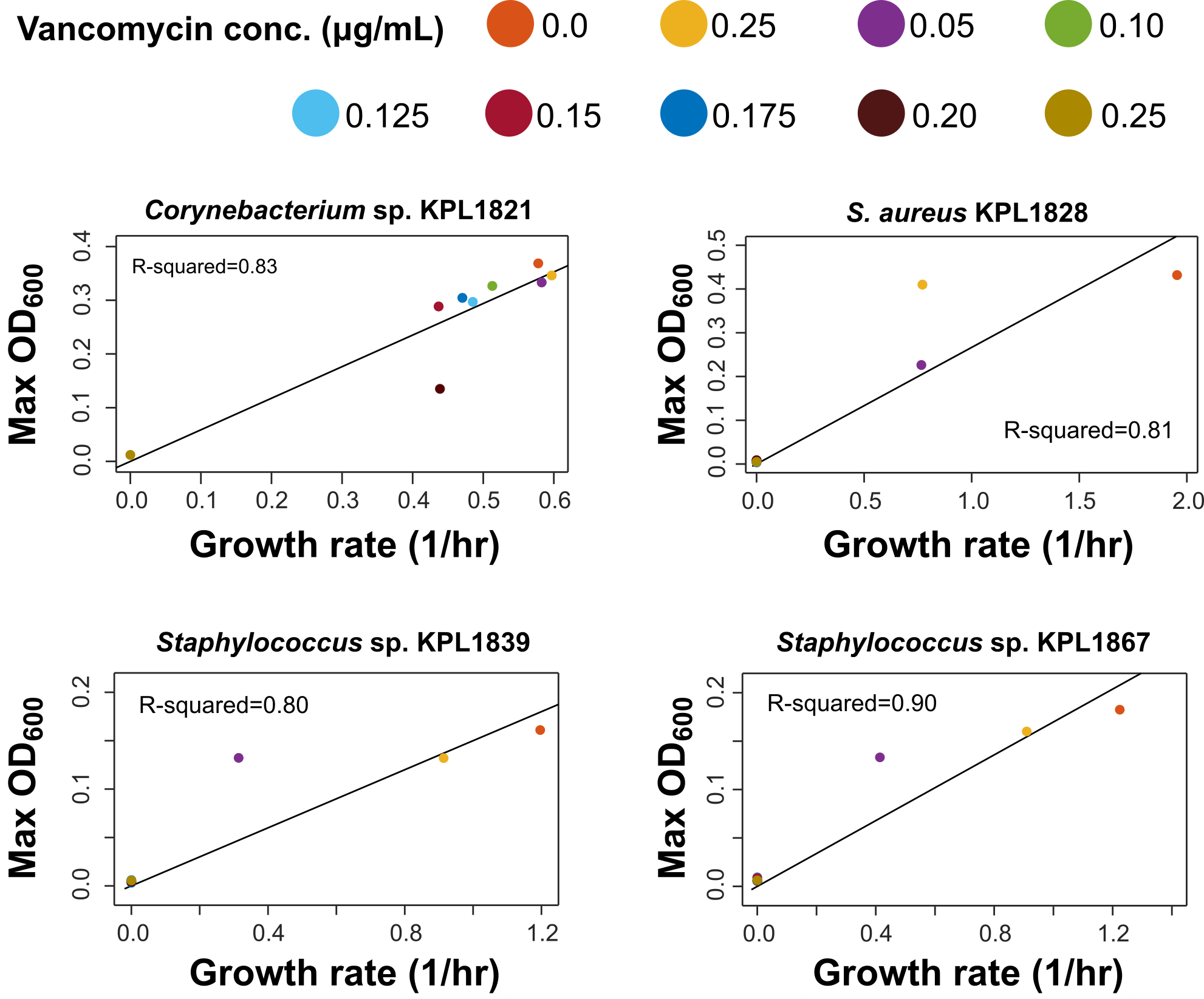

Supplement: Fig S8 — Growth rate-carrying capacity results from nasal isolates grown in the presence of vancomycin show strong correlations. Each data point shows the average growth rate and carrying capacity (using Max OD600 as a proxy) from 3 to 6 technical replicates. [file msystems.00757-22-s0008.tif]

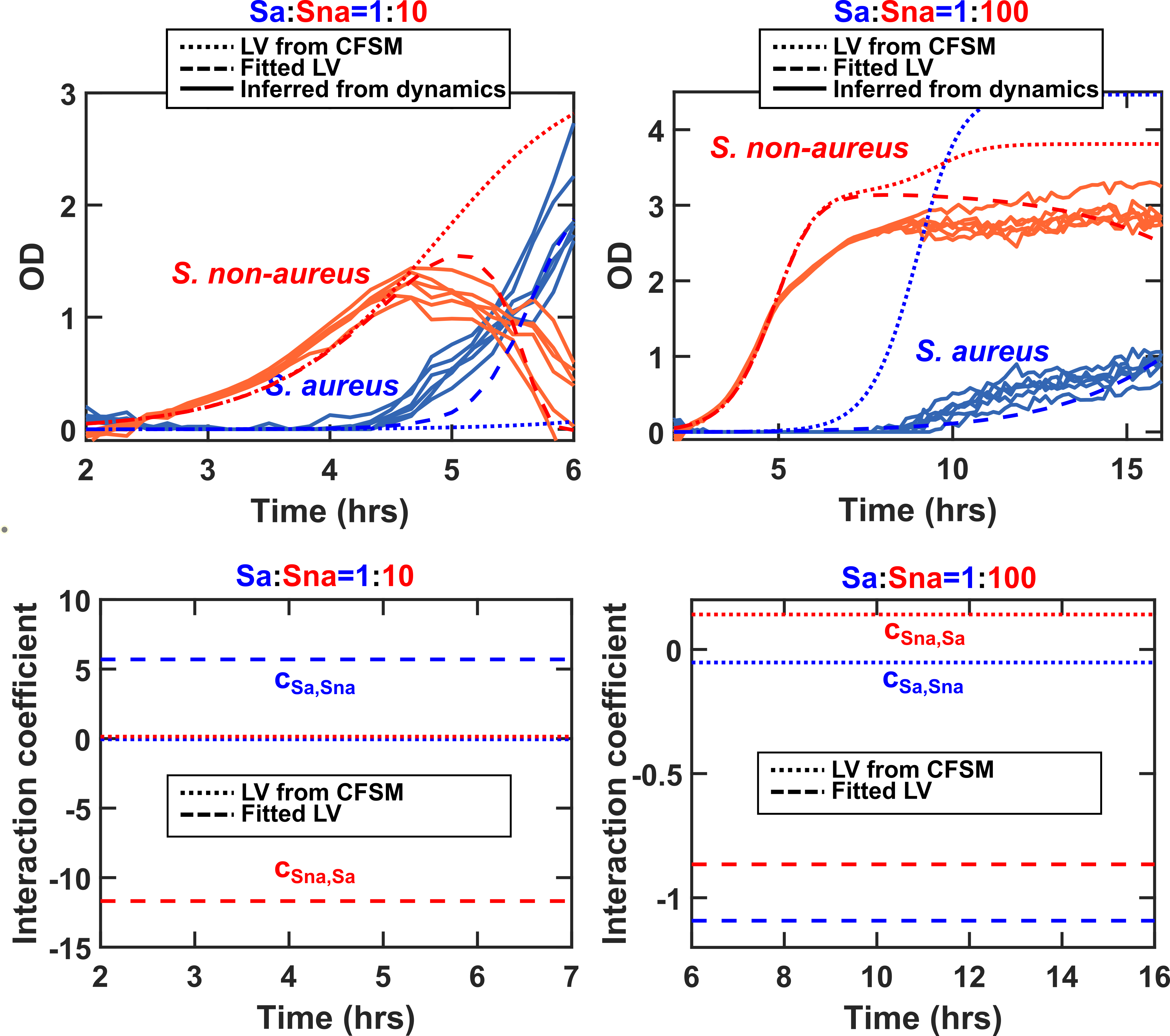

Supplement: Fig S9 — Comparison of experimental and modeling results for sGFP Staphylococcus aureus Newman (Sa) and non-aureus Staphylococcus sp. KPL1850 (Sna) cocultures shows that LV models fails to offer an acceptable approximation in rich media. Top: Coculture experiments and simulations were performed as described in the Materials and Methods section. Compared to 10% THY in Fig. 5, here 100% THY is used as the growth medium. Since S. aureus dominates these communities, initial ratios of 1:10 and 1:100 are used in this case (compared to 1:1 and 1:10 in Fig. 5). An LV model obtained from CFSM (top, dotted; bottom, dotted vs. dashed) fails to predict important trends in the dynamics. Directly fitting an LV model allows a reasonable approximation of the dynamics (top, dashed); however, the model parameters vary greatly when the initial ratio is changed, suggesting that a consistent LV model to represent the dynamics does not exist. Six technical replicates are used in each case. [file msystems.00757-22-s0009.tif]

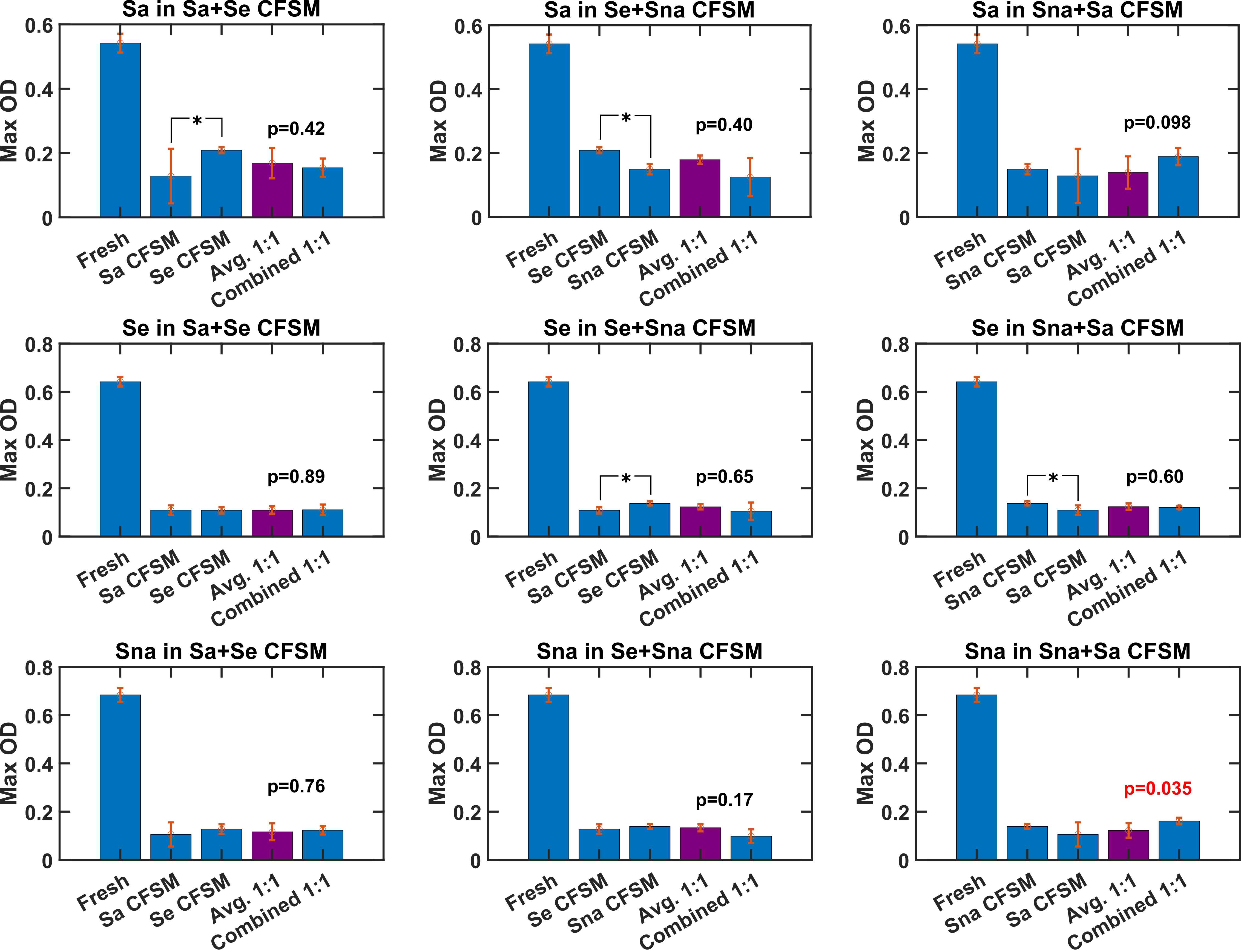

Supplement: Fig S10 — The impact of combined mixture of CFSMs of isolates is often similar to combined impact of each of the CFSMs. We tested sGFP Staphylococcus aureus (Sa), non-aureus Staphylococcus sp. KPL1839 (Se), and non-aureus Staphylococcus sp. KPL1850 (Sna) in the CFSM of each other as well as in the 1:1 combined CFSM of pairs of isolates. Our simple model predicts that the carrying capacity in the combined 1:1 CFSM (last bar in each plot) should be similar to the average of carrying capacity in each of those CFSMs (purple bar). Cases where the growth of isolates in each of the two CFSMs showed different carrying capacities are marked with an asterisk (*, T test). P values shown on each plot are for the comparison between the average 1:1 carrying capacity and the combined 1:1 carrying capacity (T test). Only in one out of the nine cases tested, the 1:1 carrying capacity and the combined 1:1 carrying capacity were different (Sna in Sna+Sa). In all cases, the culture medium was 10% THY. Error bars are standard deviation among three technical replicates. [file msystems.00757-22-s0010.tif]
